# Impaired small fiber conduction in patients with Fabry disease: a neurophysiological case–control study

**DOI:** 10.1186/1471-2377-13-47

**Published:** 2013-05-24

**Authors:** Nurcan Üçeyler, Ann-Kathrin Kahn, Daniela Kramer, Daniel Zeller, Jordi Casanova-Molla, Christoph Wanner, Frank Weidemann, Zaza Katsarava, Claudia Sommer

**Affiliations:** 1 Department of Neurology, University of Wurzburg, Josef-Schneider-Str. 11, 97080 Wurzburg, Germany; 2 Wurzburg Fabry Center for Interdisciplinary Therapy (FAZIT), Wurzburg, Germany; 3 Department of Internal Medicine I, University of Wurzburg, Wurzburg, Germany; 4 Department of Neurology, University of Essen, Essen, Germany; 5 Current address: Department of Neurology, Protestant Hospital of Unna, Unna, Germany

**Keywords:** Fabry disease, Pain-related evoked potentials, Small fiber neuropathy, A-delta fibers

## Abstract

**Background:**

Fabry disease is an inborn lysosomal storage disorder which is associated with small fiber neuropathy. We set out to investigate small fiber conduction in Fabry patients using pain-related evoked potentials (PREP).

**Methods:**

In this case–control study we prospectively studied 76 consecutive Fabry patients for electrical small fiber conduction in correlation with small fiber function and morphology. Data were compared with healthy controls using non-parametric statistical tests. All patients underwent neurological examination and were investigated with pain and depression questionnaires. Small fiber function (quantitative sensory testing, QST), morphology (skin punch biopsy), and electrical conduction (PREP) were assessed and correlated. Patients were stratified for gender and disease severity as reflected by renal function.

**Results:**

All Fabry patients (31 men, 45 women) had small fiber neuropathy. Men with Fabry disease showed impaired cold (p < 0.01) and warm perception (p < 0.05), while women did not differ from controls. Intraepidermal nerve fiber density (IENFD) was reduced at the lower leg (p < 0.001) and the back (p < 0.05) mainly of men with impaired renal function. When investigating A-delta fiber conduction with PREP, men but not women with Fabry disease had lower amplitudes upon stimulation at face (p < 0.01), hands (p < 0.05), and feet (p < 0.01) compared to controls. PREP amplitudes further decreased with advance in disease severity. PREP amplitudes and warm (p < 0.05) and cold detection thresholds (p < 0.01) at the feet correlated positively in male patients.

**Conclusion:**

Small fiber conduction is impaired in men with Fabry disease and worsens with advanced disease severity. PREP are well-suited to measure A-delta fiber conduction.

## Background

Fabry disease (FD) is an inborn lysosomal storage disorder with X-linked inheritance. Mutations in the gene encoding the enzyme α-galactosidase A (α-GAL) lead to a reduction or a complete loss of function of this key enzyme in cleavage of glycoconjugates. The consequence is the accumulation of globotriaosylceramide (Gb3) in tissues including kidneys, heart, and the nervous system [1]. FD mostly affects the peripheral nervous system in terms of small fiber neuropathy [2-5] with the main clinical feature of burning pain at palms and soles upon exertion, fever or during hot temperatures [6].

A-delta and C-fiber function can be assessed by neurological examination and quantitative sensory testing (QST). Both methods require high patient cooperation. For a more objective assessment of pathways fed by A-delta and C-fibers, specific stimulation and recording techniques are needed. Besides laser and heat stimuli, electrical current using special concentric electrodes [7] is suitable to stimulate A-delta fibers. This type of evoked potentials, which has been named “pain-related evoked potentials” (PREP), is an easily applicable new tool for objective small fiber diagnostics [8].

Here we used PREP in patients with FD to correlate A-delta fiber conduction with function and morphology. We hypothesized that small fiber neuropathy in FD should be associated with changes in A-delta pathways that are detectable by PREP recordings and asked whether these changes are associated with disease severity.

## Methods

### Subjects

We included 76 consecutive Fabry patients in this mono-center case–control study (median age 43 years, range 16–73) in whom the diagnosis of FD was confirmed by measurement of α-GAL activity in leucocytes (http://www.metabolic-genetic-disease.gmxhome.de; Munich, Germany) and genetically ascertained. The patients were prospectively recruited (2009–2011) through the Wurzburg Fabry Center for Interdisciplinary Therapy (FAZIT), University of Wurzburg. FAZIT is a tertiary referral center where patients are seen from all over Germany to confirm the diagnosis and to initiate treatment. The cohort included 31 men (median age 39 years, 16–62) and 45 women (median age 43 years, 18–73). Thirty-four patients (24 men, 10 women) were on enzyme replacement treatment (ERT; biweekly infusions of agalsidase beta [1 mg/kg body weight] in 29 patients and of agalsidase alpha [0.2 mg/kg body weight] in 5 patients). The median time on ERT at study enrolment was 4.7 years (range 0.1-9.3 years). QST and skin punch biopsy data of 66 subjects from our patient cohort were part of a data set published earlier [5].

We compared our data with data of age- and gender-matched healthy controls as detailed below. Inclusion criteria for healthy controls were: ≥16 years, no FD, no neuropathy, no neuropathic pain or other sources of pain, normal sural nerve conduction.

The study was approved by the Wurzburg Medical School Ethics Committee and was conducted in accordance with the Declaration of Helsinki. Written informed consent was obtained from all study participants.

### Clinical examination, questionnaire assessment, and laboratory tests

All patients underwent thorough neurological examination and were assessed using pain questionnaires: the German version of the Neuropathic Pain Symptom Inventory (NPSI) [9,10] and the Graded Chronic Pain Scale (GCPS), modified to a four-week recall [11]. The NPSI investigates pain intensity and characteristics resulting in a sum score between 0 (no pain at all) and 1 (maximum pain). The 24-hour recall version was used. From the GCPS we used the total score of the three pain intensity items as an indicator of pain severity, and the total score of the three items rating interference with social, occupational, and recreational activities as a disability score. To address depressive symptoms we used the German version of the Center for Epidemiologic Studies Depression Scale (“Allgemeine Depressionsskala”, ADS) [12]. The ADS ranges from 0 to 60; a score ≥ 16 is assumed clinically significant. The one week recall version was used. To make the diagnosis of small fiber neuropathy, clinical presentation, QST findings, and intraepidermal nerve fiber density (IENFD) were assessed according to Lacomis [13] (methods description see below). Depending on the number of pathological findings, definite, probable, and possible small fiber neuropathy was diagnosed. Laboratory tests included whole blood and differential cell counts; C-reactive protein; serum electrolytes; renal, liver, and thyroid function tests; erythrocyte sedimentation rate. Renal function was assessed by determining the glomerular filtration rate (GFR). Renal function, as one indicator of FD severity, was considered normal if GFR ≥ 60 ml/min/1.73 m^2^ and reduced if GFR < 60 ml/min/1.73 m^2^[14-16].

The right sural nerve was investigated in all study participants using surface electrodes and following a standard procedure [17] to exclude large fiber neuropathy. The results were compared with laboratory normal values: for antidromic sural nerve sensory nerve action potential (SNAP) amplitude ≥ 10 μV for age < 65 years, ≥ 5 μV for age > 65 years; for sural nerve conduction velocity (NCV) > 40 m/s for all ages.

### Quantitative sensory testing (QST)

QST was performed using a calibrated device (Somedic, Hörby, Sweden) and following a standardized procedure [18]. For individual analysis patients` data were compared with published reference values [19]. For group analysis patients` data were compared with values of age- and gender-matched healthy controls. All subjects were investigated at the left dorsal foot. Based on the log transformed raw values for each QST item a z-score sensory profile was calculated as follows: z-score = (value of the subject – mean value of controls)/standard deviation of controls. Negative z-scores indicate loss of sensation, positive z-scores indicate gain of sensation. We determined cold and heat detection thresholds (CDT, HDT) and the ability to detect temperature changes (thermal sensory limen, TSL) as small fiber functions. Paradoxical heat sensation (PHS) was recorded if the subject experienced cold as heat. We additionally determined the vibration detection threshold (VDT) as large fiber function. The control group for QST measurements consisted of 76 age- and gender matched healthy volunteers (45 female, 31 male; median age: 44 years, 16–73). The difference in age between patients and matched controls was three years at maximum.

### Pain-related evoked potentials (PREP)

PREP were recorded as previously described [20]. PREP were elicited by consecutive stimulation at the right and left side from face (above eyebrow), hands (medial phalanx second and third digit), and feet (dorsum) using superficial planar concentric electrodes (Inomed Medizintechnik GmbH, Lübeck, Germany) and a stimulator (Digitimer DS7A, Welwyn Garden City, UK). The potentials were recorded from Cz by a subcutaneously placed needle electrode referred to linked earlobes (A1 - A2) of the international 10–20 system using Signal Software (Version 2–16; Cambridge Electronic Design, Lt., UK). For stimulation 20 triple pulses with an intensity of two-fold of the individual pain threshold, duration of 0.5 ms, and random inter-stimulus interval of 15 to 17 seconds were applied. The potentials were recorded using the following setting: gain: x 5000, bandwidth: 1 Hz-1 kHz, sweep length: 400 ms, digitalization sampling rate: 2.5 kHz. The individual pain threshold was determined by stimulation of the area of interest twice with increasing and decreasing current intensities until the subject reported a pin-prick sensation. The average value was calculated as the individual pain threshold. Two sets of averaged curves (from n = 10 single sweeps each) were investigated for reproducible N1- (i.e. first negative peak), P1- (i.e. subsequent positive peak) latencies and peak-to-peak amplitudes (PPA) using MATLAB software (Version 7.7.0.471, The Math Works, Ismaning, Germany). All records were individually evaluated to exclude technical or biological artifacts by the same investigator who was blinded as for the diagnosis: data were assessed off-line using coded files. The control group for PREP recordings consisted of 65 healthy controls (41 female, 25 male; median age: 45 years, 21–75). Subjects with cardiac pacemakers or with seizures in their medical history were excluded.

### Skin biopsies

For assessment of intraepidermal nerve fiber density (IENFD) 5-mm skin specimens were obtained (punch device by Stiefel, Offenbach, Germany). Biopsies were taken from the lower leg (10 cm above the lateral malleolus) and from the back (at th5 level). Skin samples were processed as described previously [21]. They were immunoreacted with antibodies to protein-gene product (PGP) 9.5 (Ultraclone, UK, 1:800; primary antibody) with goat anti-rabbit IgG labelled with cyanine 3.18 fluorescent probe (Amersham, USA, 1:100; Cy3, secondary antibody), and IENFD were quantified by an observer blinded to the identity of the specimen following published rules [22]. As reference value we took data of a cohort of normal samples collected in our laboratory: lower leg: n = 110 (63 females, 47 males), median age: 50 years (range 20–84), median IENFD 7 fibers/mm, range 1–15 fibers/mm; back: n = 42 (23 female, 19 males), median age: 50, range 20–81, median IENFD 22 fibers/mm, range 6–40 fibers/mm.

### Statistical analysis

We used IBM SPSS Statistics Version 20.0 (IBM, Ehningen, Germany) for statistical analysis and creation of graphs. Non-normally distributed data were compared with the non-parametric Mann–Whitney test. Data with normal distribution were analyzed using one-way ANOVA. Data distribution was tested with the Kolmogorov-Smirnov-test and by observing data histograms. Results of non-normally distributed data are given as median and range and are illustrated as box plots. Results of normally distributed data are given as mean +/− standard deviation and are illustrated as bar graphs. For correlation analyses we used the bivariate Spearman correlation. P-values < 0.05 were considered significant.

## Results

### Clinical data, questionnaire results, and laboratory findings

Table 1 gives baseline data of the patient population. Neurological examination was normal in 44/76 cases (58%). The pathological findings mainly in the peripheral nervous system are summarized in Table 1. Neurophysiological assessment of the sural nerve revealed normal SNAP and NCV in 71/76 (93%) cases; in four patients a slight reduction in SNAP and NCV was found and in one case no potential could be obtained as a sign of sensory polyneuropathy. In none of the patients motor symptoms indicative of neuropathy were present. As for the presence of small fiber neuropathy: 16 patients had definite (11 men, 5 women), 35 patients had probable (17 men, 18 women), and 25 patients had possible small fiber neuropathy (3 men, 22 women). Assessment of pain using the NPSI did not show any intergroup differences, as almost all patients reported on “no pain” in the last 24 hours (data not shown). The GCPS (4-week recall) revealed higher pain intensities in FD patients (p < 0.001 for both genders) and higher disability scores due to pain (p < 0.05 for males, p < 0.001 for females) compared to healthy controls (Additional file 1: Figure S1a). Forty-three patients did not take analgesic medication; 19 patients used analgesic drugs on demand (n = 8: acetaminophen; n = 5: aspirin; n = 4: tramadol; n = 1 each: tilidine, ibuprofen, metamizol); twelve patients were on regular analgesic medication (n = 4: pregabalin; n = 2: gabapentin; n = 2: metamizol; n = 1 each: flupirtine, carbamazepine, tilidine, tramadol). The ADS showed higher scores for male and female FD patients compared to controls (p < 0.001 each, Additional file 1: Figure S1b). None of the patients was on antidepressant therapy. In 15/76 (20%) cases renal function was impaired; five male patients were on dialysis.

**Table 1:** Demographic and clinical characteristics of Fabry patients at baseline

	**Baseline**
FD patients (N)	76
M, F (N)	31, 45
Median age (range)	43 (16–73) years
Patients on ERT N (%)	
- M	24/31 (77%)
- F	10/45 (22%)
Median duration of ERT (range)	4.7 (range 0.1-9.3) years
Patients with GFR ≥60 or <60ml/min/1.73 m^2^ N (%)	
- M ≥60	20/31 (65%)
- M <60	11/31 (35%)
- F ≥60	41/45 (91%)
- F <60	4/45 (9%)
Findings in neurological examination N (%)	
- normal	44/76 (58%)
- hypoesthesia (thermal or tactile)	17/76 (22%)
- central pattern (e.g. hemiparesis, Babinski sign, brisk reflexes)	8/76 (11%)
- loss of ankle reflex	4/76 (5%)
- hypo- or anacusis	4/76 (5%)
- tactile hyperesthesia	2/76 (3%)

**Abbreviations**: *ERT* enzyme replacement therapy, *F* female, *FD* Fabry disease, *GFR* glomerular filtration rate, *M* male, *N* number.

### Male FD patients have impaired thermal perception

**Figure 1:**
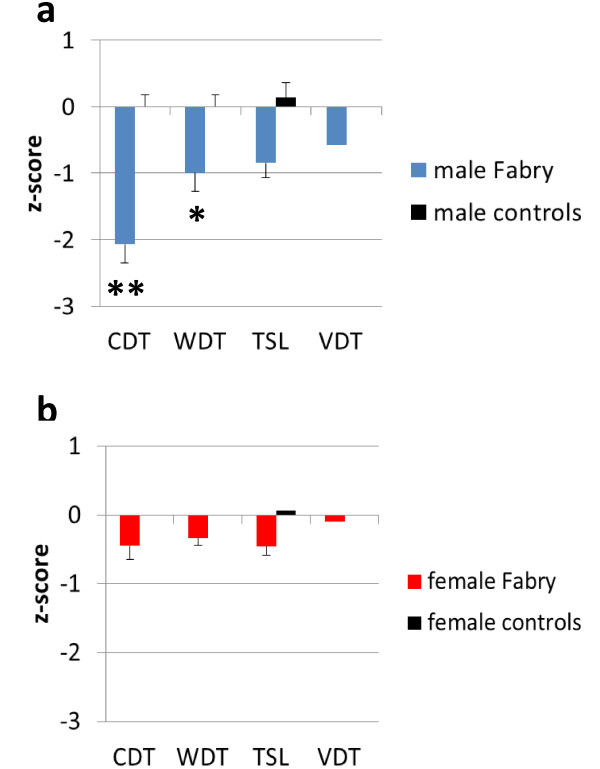
**Sensory profile of Fabry patients.** The bar graphs show the z-score sensory profiles of quantitative sensory testing (QST) at the left dorsal foot in Fabry patients compared to healthy controls. Healthy controls are represented by the black zero line. Z-scores < 0 display loss of function, z-scores >0 show gain of function. **a**) Male Fabry patients have elevated detection thresholds for cold and warm (CDT, WDT), while the thermal sensory limen (TSL) for changing temperatures and the vibration detection threshold (VDT) was not different from controls. **b**) Female Fabry patients do not differ from female controls except for slightly elevated WDT. *p < 0.05, **p < 0.01.

In line with previous reports [4,5,23] we found elevated CDT (p < 0.01) and WDT (p < 0.05) in male Fabry patients compared with male controls (Figure 1a). Female patients did not differ from female controls (Figure 1b). Male patients had higher thermal perception thresholds and impaired vibration sense compared to female FD patients (CDT: p < 0.05, WDT: p < 0.001, TSL: p < 0.01, VDT: p < 0.01). PHS was present in none of the male patients and in 9/45 (20%) female FD patients. As shown earlier male patients with impaired renal function (GFR < 60 ml/min/1.73 m^2^) had most severe impairment in thermal perception (Additional file 2: Figure S2). Women with reduced α-GAL activity did not differ in QST parameters from women with normal enzyme activity.

### Male Fabry patients have reduced PREP amplitudes

**Figure 2:**
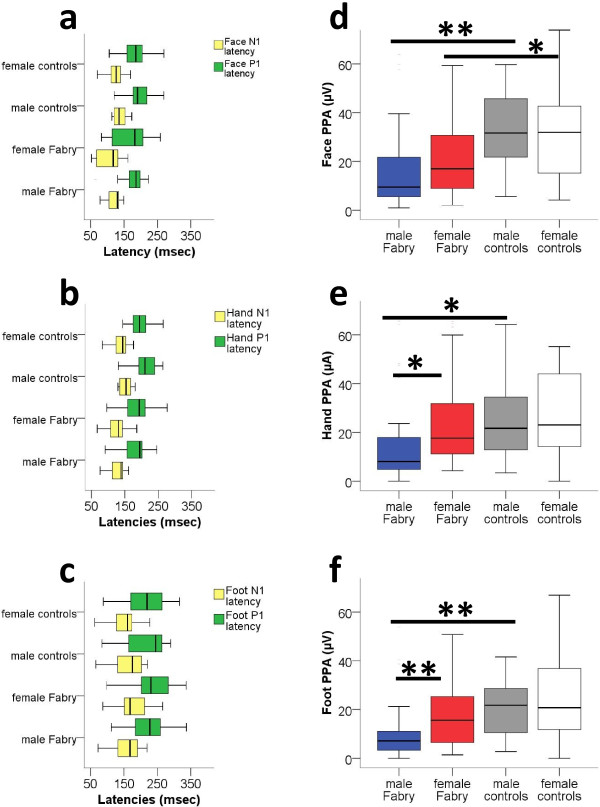
**Pain-related evoked potentials from face, hands, and feet stratified for gender.** Pain-related evoked potentials (PREP) in patients with Fabry disease and in healthy controls. **a**, **b**, **c**: N1 and P1 latencies of Fabry patients are not different from controls after eliciting PREP at the face, hand, and feet. **d**, **e**, **f**: Peak-to-peak amplitudes (PPA) of PREP are reduced in male Fabry patients when PREP is elicited at the face, the hands, or the feet. *p < 0.05, **p < 0.01.

**Figure 3:**
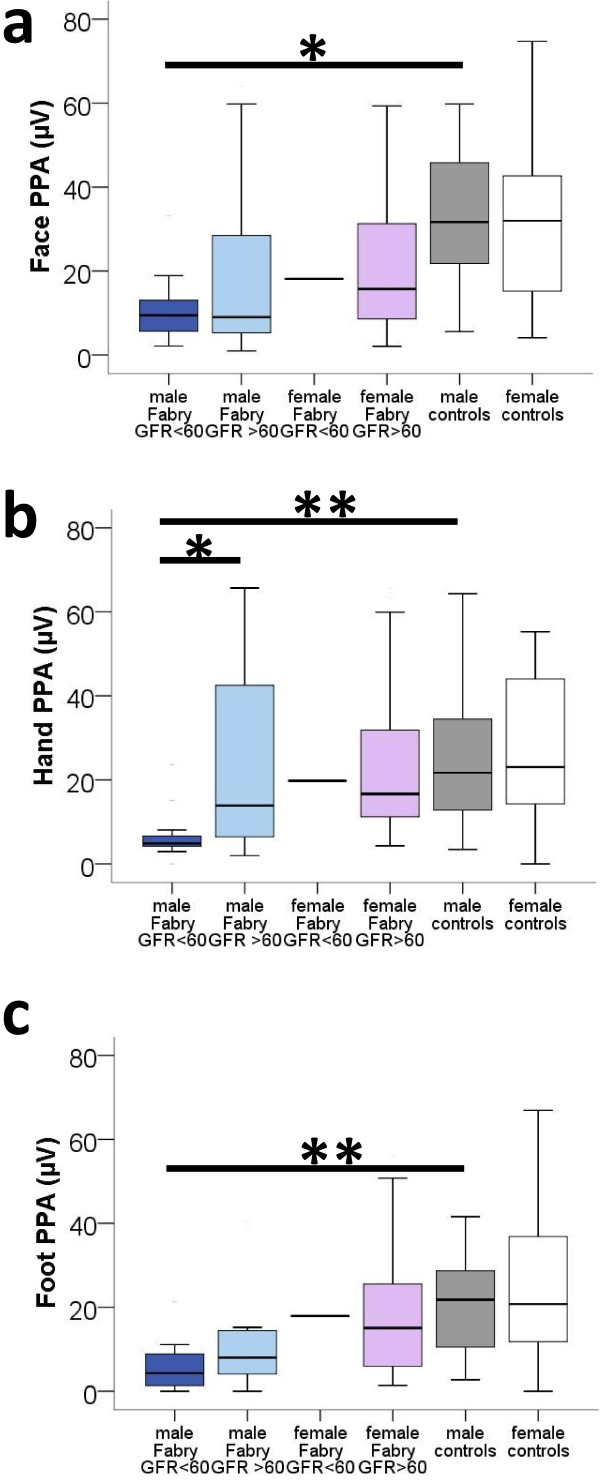
**Pain-related evoked potential amplitudes from face, hands, and feet stratified for gender and disease severity.** Boxplots show the comparison of PREP PPA stratified for renal function. Male Fabry patients with impaired renal function have reduced PREP PPA after eliciting PREP from face (p = 0.012, **a**), hands (p = 0.007, **b**), and feet (p = 0.007, **c**). *p < 0.05, **p < 0.01.

PREP data from both sides were pooled, since there was no difference between the right and left side for any subject group, investigated area, or PREP parameter. Stimulus intensities did not differ between groups for all three areas. PREP N1 and P1 latencies did not differ between FD patients and controls (Figure 2a-c). Peak-to-peak amplitudes (PPA), however, were lower in male Fabry patients compared to male controls upon stimulation at face (p < 0.01), hands (p < 0.05), and feet (p < 0.01, Figure 2d-f). Male Fabry patients also had lower PPA compared to female patients (hand: p < 0.05, foot: p < 0.01, Figure 2d-f). When comparing data of male Fabry patients with GFR < 60 and GFR > 60 with data of healthy male controls, only patients with impaired renal function showed reduced PPA after eliciting PREP from face (p = 0.012), hands (p = 0.007), and feet (p = 0.007, Figure 3a-c). Stimulus intensities did not differ between patients and controls at any site. Women with reduced α-GAL activity did not differ from women with normal enzyme activity as for PREP parameters. Additional file 3: Figure S3 shows an example of a PREP recording after stimulation at the face.

### Male Fabry patients with advanced disease have the most marked reduction in skin innervation

Twenty male patients (20/31, 65%) and 27/45 female patients (60%) agreed to skin punch biopsy. In line with previous findings [5] we found reduced distal and proximal IENFD in male patients compared to healthy controls. Fiber reduction was most prominent in male patients with impaired renal function: distal (p < 0.001) and proximal (p < 0.05) IENFD was lower compared to male healthy controls (Additional file 4: Figure S4). Similarly, female patients with reduced renal function had lower distal IENFD than female controls (p < 0.05, Additional file 4: Figure S4a), while skin innervation was not different at the back (Additional file 4: Figure S4b). Lower GFR correlated with lower distal IENFD (correlation coefficient: 0.508, p < 0.001) and with lower proximal IENFD (correlation coefficient: 0.369, p < 0.05). Women with reduced α-GAL activity did not differ from women with normal enzyme activity as for skin innervation.

### PREP amplitudes correlate with impaired thermal perception in Fabry patients

**Figure 4:**
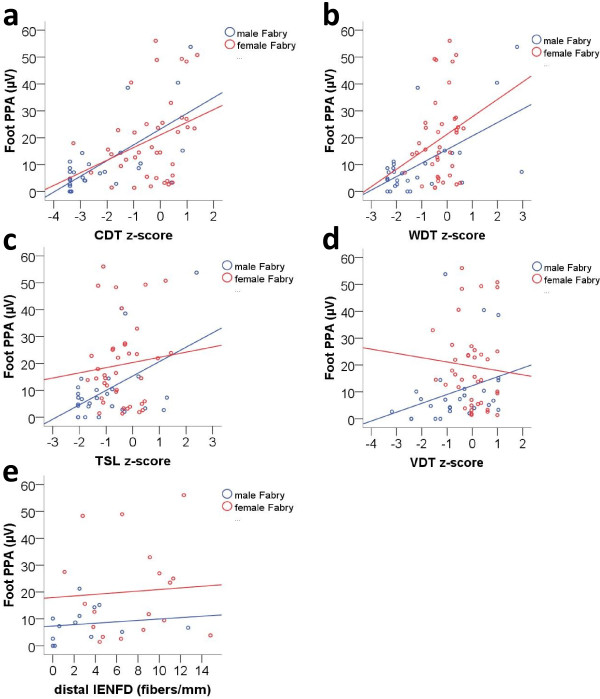
**Correlations of sensory profile and nerve fiber density with pain**-**related evoked potential amplitudes.** Scatter plots show correlations between PREP peak-to-peak-amplitudes (PPA) recoded after stimulation at the feet and QST parameters (warm detection threshold, WDT; cold detection threshold, CDT; thermal sensory limen, TSL; vibration detection threshold, VDT). **a**) PREP PPA correlated positively with CDT in male (correlation coefficient: 0.564, p = 0.002) and female Fabry patients (correlation coefficient: 0.340, p = 0.034). **b**) PREP PPA correlated positively with WDT only in male Fabry patients (correlation coefficient: 0.390, p = 0.04). **c**, **d**) TSL and VDT did not correlate with PREP PPA in either gender. **e**) Distal IEFND did not correlate with PREP PPA obtained after stimulation at the foot.

A positive correlation was found between PREP PPA elicited at the feet and thermal perception thresholds (CDT, WDT) determined at the feet of Fabry patients (Figure 4a, b). In male patients a positive correlation was found for CDT (correlation coefficient: 0.564, p < 0.01) and for WDT (correlation coefficient: 0.390, p < 0.05). In female patients a positive correlation was found only for CDT (correlation coefficient: 0.340, p < 0.05). TSL and VDT did not correlate with PREP PPA in both genders (Figure 4c, d). Also, distal IENFD did not show a correlation with PREP parameters obtained after stimulation at the feet (illustrated for PPA in Figure 4e).

## Discussion

We prospectively investigated a large cohort of consecutive Fabry patients with small fiber neuropathy using electrical stimulation of A-delta fibers (PREP), and compared the findings with complementary methods for small fiber function and morphology, namely QST and histological assessment of skin innervation. We show that A-delta fiber conduction can easily be assessed using PREP, that A-delta fiber conduction is impaired mainly in male Fabry patients with advanced disease, and that pathological PREP parameters correlate with reduced thermal perception.

Elevated cold detection thresholds are the most marked clinical finding when examining the peripheral nervous system in male Fabry patients [2,4,5,23-28]. Innocuous cold sensation is mostly an A-delta fiber function with some C-fiber participation [29]. PREP allows the activation of A-delta afferents in superficial skin layers by the high current intensity when applying low current stimulation. This is achieved by the usage of concentric electrodes that have a small anode–cathode distance. The electrical stimulation leads to a pin-prick sensation, and nerve conduction velocities of fibers stimulated by such concentric electrodes are in range with those of A-delta fibers [20], [30-32]. Using PREP, we found a reduction in evoked potential amplitudes in male Fabry patients which was mostly due to the subgroup with advanced disease. The finding of reduced PREP amplitudes is consistent with data from studies investigating patients with small fiber pathology in diabetes [32], HIV infection [33], and fibromyalgia syndrome [20]. Moreover, PREP amplitudes positively correlated with cold and warm perception as measured with QST mainly in male Fabry patients. This underscores the notion that small fiber neuropathy in FD leads to A-delta impairment. This finding is also supported by the only previous neurophysiological study on small fibers in FD, which used laser evoked potentials (LEP) in seven male Fabry patients and found lower A-delta LEP amplitudes compared to controls [34].

The reason for A-delta fiber dysfunction in FD is not fully understood. One hypothesis is that the peripheral nerve fibers degenerate due to Gb3 deposits in dorsal root ganglion (DRG) neurons. Such deposits have been detected in histopathological studies in Fabry patients [35]. Accumulation of Gb3 in DRG may lead to neuronal apoptosis with a dying back neuropathy in terms of a ganglionopathy and may result in reduced IENFD. This fits well to the observed general reduction of intraepidermal nerve fibers in Fabry patients also in skin from the back, which normally is preserved from intraepidermal fiber loss in peripheral neuropathies spreading from distal to proximal. The reason why small fibers are more vulnerable is unknown. Gb3 deposition in neurons of DRG may also lead to neuronal dysfunction e.g. by altering cellular excitability on the basis of ion channel alterations. This has been shown for endothelial potassium channels in the Fabry mouse model [36]. As a consequence sensory impairment with clinically observed thermal hypoesthesia may occur.

The concept of Gb3 deposits as a basis of the progressive deterioration in small fiber conduction, function, and morphology mainly in men with advanced disease severity is plausible because hemizygote men have a higher Gb3 load and Gb3 deposition increases with time. Moreover, Gb3 clearance by ERT in DRG neurons may be hampered by the blood-brain-barrier possibly resulting in deterioration of cold detection thresholds even under treatment [5].

One limitation of our study is that we had to recruit individual control groups for QST, PREP, and skin punch biopsy. The majority of the control subjects refused to take part in all three study sections (QST for one hour, PREP for another hour, skin punch biopsy for another 20 minutes and biopsy at two body sites). However, strict in- and exclusion criteria were observed and patients and controls were matched as for gender and age.

## Conclusions

Our study gives evidence for A-delta nerve fiber impairment mainly in male Fabry patients with advanced disease severity and show that PREP measurements are an easily applicable, robust, and objective method to investigate A-delta carried sensory pathways.

## Abbreviations

ADS: “Allgemeine Depressionsskala”; α-GAL: alpha-galactosidase A; CDT: Cold detection threshold; ERT: Enzyme replacement therapy; FD: Fabry disease; Gb3: Globotriaosylceramide3; GCPS: Graded Chronic Pain Scale; GFR: Glomerular filtration rate; NPSI: Neuropathic Pain Symptom Inventory; PPA: Peak-to-peak amplitude; PHS: Paradoxical heat sensation; PREP: Pain-related evoked potentials; QST: Quantitative sensory testing; VDT: Vibration detection threshold; WDT: Warm detection threshold.

## Competing interests

NÜ: received honoraria for consultancy from Grünenthal GmbH and for presentations from Genzyme Corp., Eczacıbaşı-Baxter, and Astellas; received travel grants from Pfizer Inc., Eczacıbaşı-Baxter, Genzyme Corp., Astellas, Grünenthal GmbH, CSL Behring, and Shire Corp.; she has received research support from Grünenthal GmbH. AKK, DK, JCM: report no disclosures. DZ: received a travel grant from UCB Pharma. CW and FW: received speaker honoraria from Genzyme and Shire Corp.; both authors are members of the Fabry Registry European Board of Advisors and have received travel assistance and speaker honoraria. Research grants were given to the Department of Internal Medicine, University of Wurzburg, by Genzyme and Shire Corps. ZK: has received grants and research support, been a consultant for, and is a member of the speakers’ bureau of Allergan, Inc. CS: has served on scientific advisory boards for Astellas Pharma Inc, Baxter Inc, Eli Lilly and Company, Genzyme, Pfizer Inc, and UCB; she serves on the editorial boards of the Journal of the Peripheral Nervous System, PAIN, PLOS ONE, Journal of Neurology, Aktuelle Neurologie, European Journal of Pain, and Schmerz; she has received speaker honoraria from Allergan, Baxter Inc., CSL Behring, Eli Lilly, Genzyme Corp, GSK and Pfizer Inc.; and she has received research support from Genzyme Corp., the German Research Foundation (SFB 581) and from the Interdisciplinary Center for Clinical Research of the University of Wurzburg.

## Authors’ contributions

NÜ drafted designed the study concept, performed the clinical examination of patients, acquired data, established PREP measurements, performed the statistical data analysis and interpreted the results, prepared the manuscript. AKK performed PREP measurements, acquired data, and assessed PREP data. DK performed the clinical examination of patients and acquired data. DZ established PREP measurements and analyzed PREP data. JCM performed PREP measurements and acquired data. CW performed the clinical examination of patients, acquired data, and revised the manuscript. FW performed the clinical examination of patients, acquired data, and revised the manuscript. ZK established PREP measurements and revised the manuscript. CS designed the study concept, performed the clinical examination of patients, acquired data, interpreted data, and prepared the manuscript. All authors read and approved the final manuscript.

## Pre-publication history

The pre-publication history for this paper can be accessed here:

http://www.biomedcentral.com/1471-2377/13/47/prepub

## Supplementary Material

Additional file 1: Figure S1 Sensory profile of Fabry patients stratified for gender and disease severity
Sensory profile of Fabry patients stratified for gender and disease severity. The bar graphs show the z-score sensory profiles of quantitative sensory testing (QST) at the left dorsal foot in Fabry patients compared to healthy controls after stratification for gender and renal function. Healthy controls are represented by the black zero line. Z-scores < 0 display loss of function, z-scores >0 show gain of function. Male Fabry patients with impaired renal function (i.e. glomerular filtration rate [GFR] < 60 ml/min/1.73 m^2^ show most impaired perception thresholds for cold and warm (CDT, WDT) and temperature changes (thermal sensory limen [TSL]). Also the vibration detection threshold (VDT) is impaired. Male patients with normal renal function (GFR ≥ 60 ml/min/1.73 m^2^) also show impairment of CDT, WDT, and TSL, however, less severe than in male patients with impaired renal function. Female patients with reduced renal function also show a tendency for impaired CDT and WDT, while women with normal renal function do not differ from controls. Click here for file

Additional file 2: Figure S2 Pain and depression questionnaire results
Pain and depression questionnaire results. Results of questionnaire assessment with the Graded Chronic Pain Scale (GCPS) and the depression scale “Allgemeine Depressionsskala” (ADS). A) Male and female Fabry patients have higher scores for pain intensity and disability due to pain in the last four weeks compared to healthy controls. B) Fabry patients reach higher scores for depressive symptoms on the ADS compared to healthy controls independent of gender. The horizontal line in the boxplots represent median values; the boxes end with the 25th and 75th quartile; the whiskers indicate the highest and lowest value. *p < 0.05, **p < 0.01, ***p < 0.001. Click here for file

Additional file 3: Figure S3 PREP record after stimulation at the face
PREP record after stimulation at the face. The upper plot shows a PREP record from Cz after triple electrical stimulation at the face (above eyebrow). The three parameters investigated were A) the N1 latency, B) the P1 latency, and C) the peak-to-peak amplitude. The lower plot shows the control record of possible blink artifacts during the stimulation at the face, which might disturbe the PREP recordings. The zero line in the illustrated case shows that no blink artifacts were present. Click here for file

Additional file 4: Figure S4 Results of intraepidermal nerve fiber density stratified for gender and disease severity
Results of intraepidermal nerve fiber density stratified for gender and disease severity. Intraepidermal nerve fiber density (IENFD) at the lower leg (a) and the back (b) of Fabry patients and of healthy controls assessed with the pan-axonal marker PGP9.5 and stratified for renal function. a + b) Male patients with impaired renal function have lower PGP9.5 positive IENFD compared to healthy controls at the lower leg and the back. Also female patients with reduced renal function have lower IENFD at the lower leg. *p < 0.05, **p < 0.01, ***p < 0.001. Click here for file
