# Supplementary figures and images for: Impaired small fiber conduction in patients with Fabry disease: a neurophysiological case–control study
Source: BMC Neurol. 2013 May 24;13:47. doi: 10.1186/1471-2377-13-47 (PMC3672069; doi:10.1186/1471-2377-13-47)

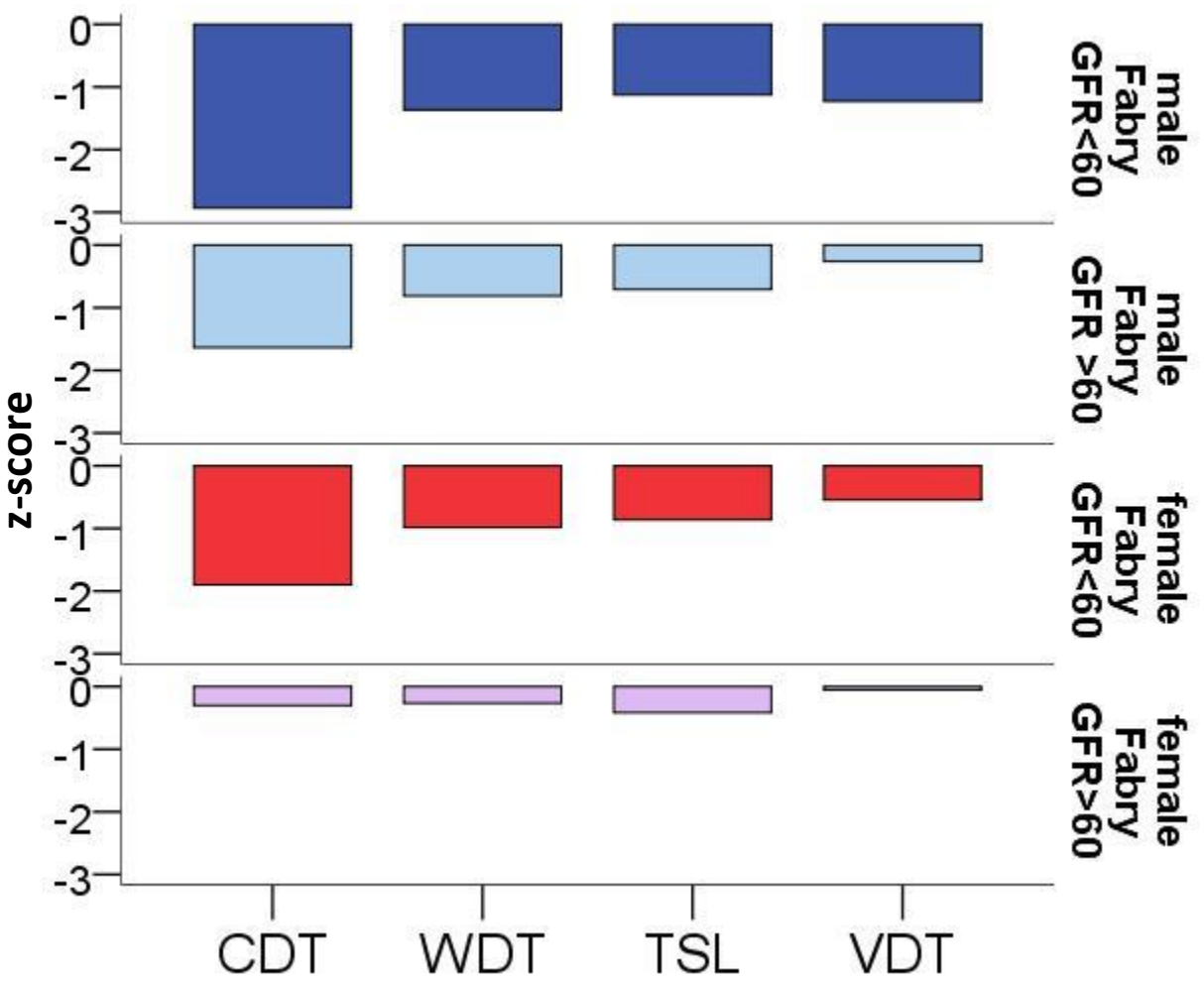

Supplement: Additional file 1: Figure S1 — Sensory profile of Fabry patients stratified for gender and disease severity Sensory profile of Fabry patients stratified for gender and disease severity. The bar graphs show the z-score sensory profiles of quantitative sensory testing (QST) at the left dorsal foot in Fabry patients compared to healthy controls after stratification for gender and renal function. Healthy controls are represented by the black zero line. Z-scores < 0 display loss of function, z-scores >0 show gain of function. Male Fabry patients with impaired renal function (i.e. glomerular filtration rate [GFR] < 60 ml/min/1.73 m2 show most impaired perception thresholds for cold and warm (CDT, WDT) and temperature changes (thermal sensory limen [TSL]). Also the vibration detection threshold (VDT) is impaired. Male patients with normal renal function (GFR ≥ 60 ml/min/1.73 m2) also show impairment of CDT, WDT, and TSL, however, less severe than in male patients with impaired renal function. Female patients with reduced renal function also show a tendency for impaired CDT and WDT, while women with normal renal function do not differ from controls. [file 1471-2377-13-47-S1.pdf]

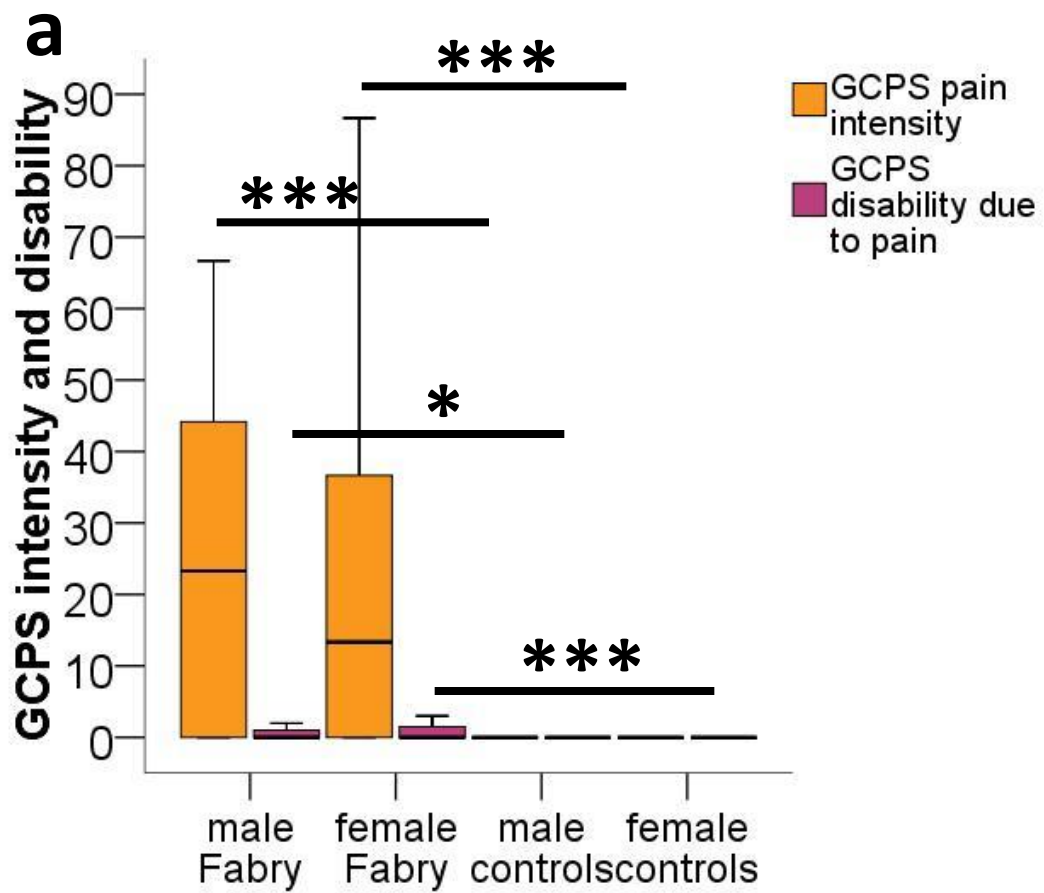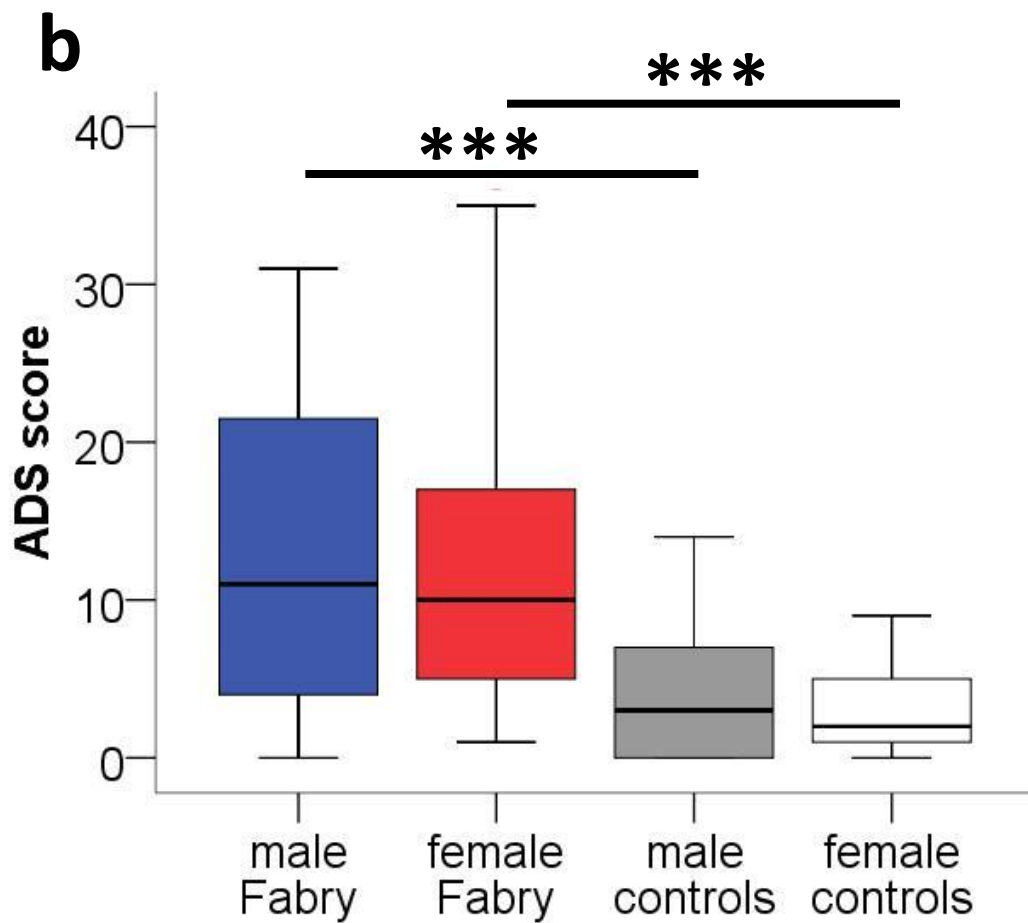

Supplement: Additional file 2: Figure S2 — Pain and depression questionnaire results Pain and depression questionnaire results. Results of questionnaire assessment with the Graded Chronic Pain Scale (GCPS) and the depression scale “Allgemeine Depressionsskala” (ADS). A) Male and female Fabry patients have higher scores for pain intensity and disability due to pain in the last four weeks compared to healthy controls. B) Fabry patients reach higher scores for depressive symptoms on the ADS compared to healthy controls independent of gender. The horizontal line in the boxplots represent median values; the boxes end with the 25th and 75th quartile; the whiskers indicate the highest and lowest value. *p < 0.05, **p < 0.01, ***p < 0.001. [file 1471-2377-13-47-S2.pdf]

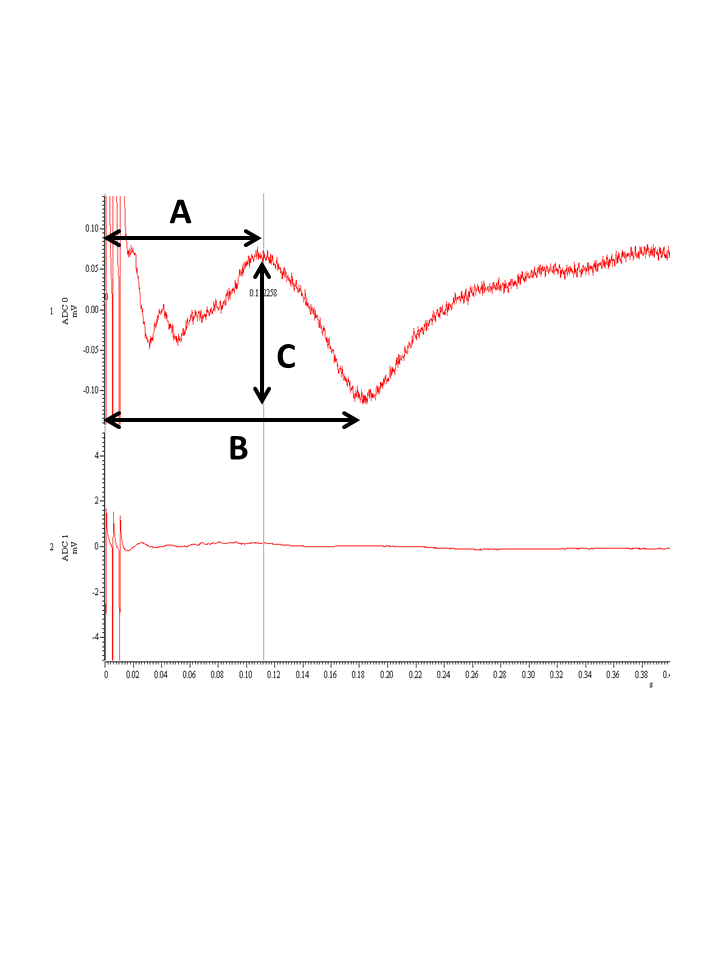

Supplement: Additional file 3: Figure S3 — PREP record after stimulation at the face PREP record after stimulation at the face. The upper plot shows a PREP record from Cz after triple electrical stimulation at the face (above eyebrow). The three parameters investigated were A) the N1 latency, B) the P1 latency, and C) the peak-to-peak amplitude. The lower plot shows the control record of possible blink artifacts during the stimulation at the face, which might disturbe the PREP recordings. The zero line in the illustrated case shows that no blink artifacts were present. [file 1471-2377-13-47-S3.tiff]

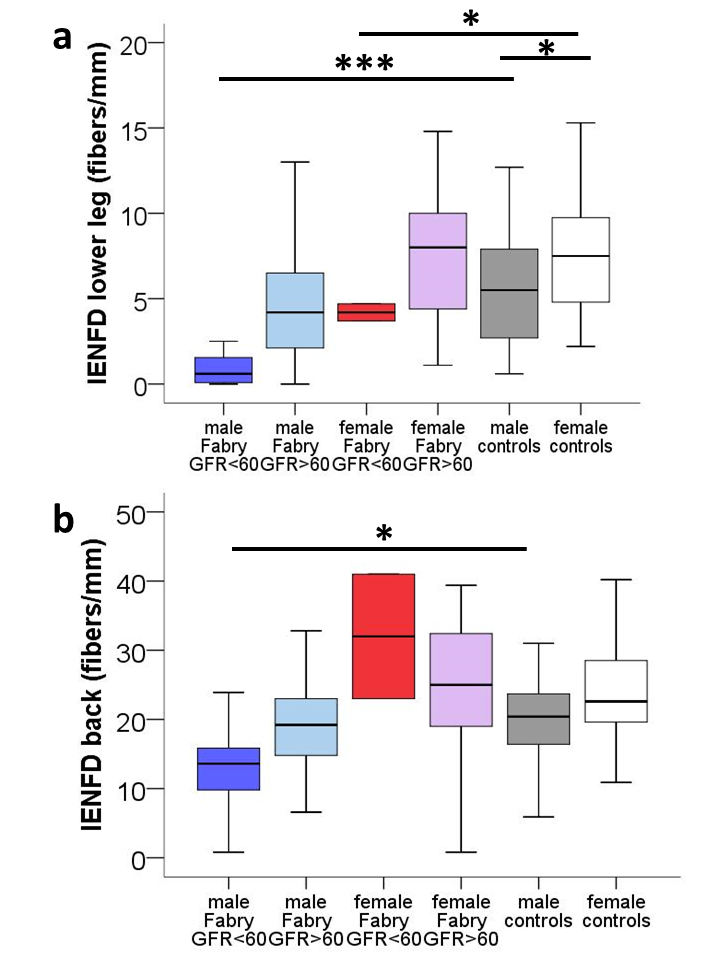

Supplement: Additional file 4: Figure S4 — Results of intraepidermal nerve fiber density stratified for gender and disease severity Results of intraepidermal nerve fiber density stratified for gender and disease severity. Intraepidermal nerve fiber density (IENFD) at the lower leg (a) and the back (b) of Fabry patients and of healthy controls assessed with the pan-axonal marker PGP9.5 and stratified for renal function. a + b) Male patients with impaired renal function have lower PGP9.5 positive IENFD compared to healthy controls at the lower leg and the back. Also female patients with reduced renal function have lower IENFD at the lower leg. *p < 0.05, **p < 0.01, ***p < 0.001. [file 1471-2377-13-47-S4.tiff]
